# Supplementary material for: Rare-event sampling of epigenetic landscapes and phenotype transitions
Source: PLoS Comput Biol. 2018 Aug 3;14(8):e1006336. doi: 10.1371/journal.pcbi.1006336 (PMC6093701; doi:10.1371/journal.pcbi.1006336)
Supplement: S1 File — (PDF) [file pcbi.1006336.s001.pdf]

Supporting Information

1 ExMISA Network

Two-gene network with Mutual Inhibition, Self-Activation, and exclusive transcription factor binding.

$$\mathrm{A_{00}} + 2\,\mathrm{a} \underset{\mathrm{f}_a}{\overset{\mathrm{h}_a}{\rightleftharpoons}} \mathrm{A_{10}}$$
$$\mathrm{A_{00}} + 2\,\mathrm{b} \underset{\mathrm{f}_r}{\overset{\mathrm{h}_r}{\rightleftharpoons}} \mathrm{A_{01}}$$
$$\mathrm{B_{00}} + 2\,\mathrm{b} \underset{\mathrm{f}_a}{\overset{\mathrm{h}_a}{\rightleftharpoons}} \mathrm{B_{10}}$$
$$\mathrm{B_{00}} + 2\,\mathrm{a} \underset{\mathrm{f}_r}{\overset{\mathrm{h}_r}{\rightleftharpoons}} \mathrm{B_{01}}$$
$$\mathrm{A_{00}} \xrightarrow{\mathrm{g_0}} \mathrm{A_{00}} + \mathrm{a}$$
$$\mathrm{B_{00}} \xrightarrow{\mathrm{g_0}} \mathrm{B_{00}} + \mathrm{b}$$
$$\mathrm{A_{01}} \xrightarrow{\mathrm{g_0}} \mathrm{A_{01}} + \mathrm{a}$$
$$\mathrm{B_{01}} \xrightarrow{\mathrm{g_0}} \mathrm{B_{01}} + \mathrm{b}$$
$$\mathrm{A_{10}} \xrightarrow{\mathrm{g_1}} \mathrm{A_{10}} + \mathrm{a}$$
$$\mathrm{B_{10}} \xrightarrow{\mathrm{g_1}} \mathrm{B_{10}} + \mathrm{b}$$
$$\mathrm{a} \xrightarrow{\mathrm{k}} 0$$
$$\mathrm{b} \xrightarrow{\mathrm{k}} 0$$

2 Pluripotency network

There are eight genes (encoding transcription factors) in the pluripotency network. Transcription factors bind as homodimers with the exception of the OCT4-SOX2 heterodimer. Only three transcription factors interact with their own gene, CDX2, NANOG, and GATA6. Transcription factors bind as dimers with the rate h and unbind with the rate f. When a gene is bound by any activator and no repressors, it expresses at a rate g_{on}, otherwise, it expresses at a rate g_{off}. The only exception is NANOG, which must be bound by all three of its activators and no repressors to be activated.
