# Supplementary material for: Rare-event sampling of epigenetic landscapes and phenotype transitions
Source: PLoS Comput Biol. 2018 Aug 3;14(8):e1006336. doi: 10.1371/journal.pcbi.1006336 (PMC6093701; doi:10.1371/journal.pcbi.1006336)
Supplement: S2 File — (PDF) [file pcbi.1006336.s002.pdf]

1 Weighted Ensemble Exploration Mode

1. Format the reaction network into a BioNetGen file.
2. Choose M_{targ}, the target number of replicas per sampling region, and N_{bins}, the target number of sampling regions or bins.
3. Initialize M_{targ} replicas in a single starting location.
4. Simulate replicas for a simulation time τ_{WE}. Replicas are simulated in parallel.
5. Chose N_{bins} new bin positions.
   (a) Chose one random replica as the first new bin position.
   (b) Chose the replica furthest from the set of bin positions to be the next new bin position.
   (c) Repeat (b) until N_{bins} new positions have been chosen.
6. Perform the WE step.
   (a) For a given bin, if the number of replicas in the bin is less than M_{targ}, split the replica with the largest weight into n equally weighted replicas until there are M_{targ} replicas.
   (b) For a given bin, if the number of replicas in the bin is greater than M_{targ}, combine the weight of n replicas and randomly chose one to receive the combined weight such that there are M_{targ} replicas in the bin.
   (c) Repeat (a) or (b) for each sampling bin
7. Repeat steps 4-6 for a chosen number of simulation steps.

2 Transition-Matrix Mode

1. Start from the end of exploration mode.
2. Simulate replicas for a time τ_{WE}.
3. Collect weights transferred from bin i to bin j over the simulation period τ into a transition matrix.
4. Perform the WE step.
5. Repeat steps 2-4 for a chosen number of simulation steps.

3 Rate-Estimation Mode

1. Start from the end of exploration mode.
2. Label replicas as having most recently visited region of interest X or visited region of interest Y.
3. Simulate replicas for a time τ_{WE}.
4. Collect weights transferred from X to Y over the simulation period τ and change the replica label as necessary.
5. (Optional) Chose N_{bins} new sampling regions.
6. Perform the WE step.
7. Repeat steps 3-6 for a chosen number of simulation steps.

4 Coarse-Graining Procedure

1. Find the left-eigenvalues and eigenvectors of the row-stochastic transition matrix calculated from transition-matrix estimation mode.
   (a) The probability distribution of the system is estimated by the left-eigenvector associated with the eigenvalue $\lambda = 1$
2. Perform the PCCA+ algorithm using MSMBuilder software to cluster the N_{bins} sampling regions into macrostates. MSMBuilder software outputs a Markov State Model of the reaction network.
3. Use transition path analysis (using PyEMMA software) on the resulting MSM to obtain parallel transition paths or estimate the rate of transitioning between any two states.
4. Gephi 0.7 is used to visualize the row-stochastic transition matrix and the MSM.
