# Supplementary material for: Rare-event sampling of epigenetic landscapes and phenotype transitions
Source: PLoS Comput Biol. 2018 Aug 3;14(8):e1006336. doi: 10.1371/journal.pcbi.1006336 (PMC6093701; doi:10.1371/journal.pcbi.1006336)
Supplement: S3 File — (PDF) [file pcbi.1006336.s003.pdf]

In order to estimate $\widetilde{\mathbf{T}}(\tau)$ from conventional simulation, we estimate transition probabilities from counts, i.e., each element of $\widetilde{\mathbf{T}}(\tau)$ is estimated by $T_{ij} = C_{ij}/C_i$, where the system is observed at intervals of τ, C_{ij} is the count of total observations of the system in state j given that it was previously observed in state i at a time τ earlier, and $C_i = \sum_j C_{ij}$ is the total number of observations in state i. It follows that

$$\Big[\text{Sim. steps to estimate } \widetilde{\mathbf{T}}(\tau), \text{ Conv.}\Big] = \tau \sum_i C_i \geq \tau \sum_i \left(\min_j\{T_{ij}\}\right)^{-1}, \tag{1}$$

where the first equality states that the total simulation time equals the sum over times spent in each bin. The second (in)equality follows from the fact that $C_{ij} \geq 1$, i.e., each nonzero element reflects a transition that was observed at least once. For a given state i, the rarest transition out of i has estimated transition probability $\min_j\{T_{ij}\}$. If the rarest transition was sampled just once, then $C_i = \left(\min_j\{T_{ij}\}\right)^{-1}$ (which also equals the expected time to observe the rarest transition out of state i).

In principle, Eq 1 provides a means of estimating a lower bound on the number of conventional simulation steps required to have achieved a given, WE-estimated $\widetilde{\mathbf{T}}(\tau)$. However, Eq 1 has the drawback of being dependent on the accuracy of the smallest T_{ij} elements (i.e., the rarest sampled transitions in the network). We found that a more conservative (and less error-prone) estimate of efficiency gain could be achieved by replacing $\min_j\{T_{ij}\}$ with $P_{5\%,i}\{T_{ij}\}$, i.e., the 5th-percentile of the nonzero sampled elements in each row. The choice of 5% showed efficiency gain estimates for ExMISA that were conservative, yet in line with results from long conventional SSA simulations.

Ideally, an efficiency metric should compare the required simulation time to achieve $\widetilde{\mathbf{T}}(\tau)$ with some common degree of accuracy, from conventional simulation versus WE. While the nature of errors associated with individual T_{ij} estimates based on our computational pipeline have not yet been thoroughly explored, for the ExMISA system it is possible to compare the estimated $\widetilde{\mathbf{T}}(\tau)$ to a theoretical benchmark (i.e., as computed from the rate matrix). For the maximum simulation time reached in the conventional simulation, the WE-simulation had run about one-third as long to reach the same level of error or lower (as measured by the 2-norm, see **??**). This number is commensurate with the estimate reached by Eq **??** in the main text of a two-fold speedup for ExMISA.
