# Supplementary material for: Rare-event sampling of epigenetic landscapes and phenotype transitions
Source: PLoS Comput Biol. 2018 Aug 3;14(8):e1006336. doi: 10.1371/journal.pcbi.1006336 (PMC6093701; doi:10.1371/journal.pcbi.1006336)
Supplement: S1 Table — (PDF) [file pcbi.1006336.s004.pdf]

**ExMISA Parameters**	**Value in** $[1/k]$	**Description**
g_0	4.0	basal/ repressed expression rate
g_1	18.0	activated expression rate
h_a	1×10^{-5}	binding rate of activator
h_r	1×10^{-1}	binding rate of repressor
f_a	1×10^{-5}	unbinding rate of activator
f_r	1	unbinding rate of repressor
k	1	transcription factor degradation rate

Table S1. ExMISA Network Parameters
