# Supplementary material for: Rare-event sampling of epigenetic landscapes and phenotype transitions
Source: PLoS Comput Biol. 2018 Aug 3;14(8):e1006336. doi: 10.1371/journal.pcbi.1006336 (PMC6093701; doi:10.1371/journal.pcbi.1006336)
Supplement: S2 Table — (PDF) [file pcbi.1006336.s005.pdf]

Gene	Activators	Repressors
$PBX1$	NANOG	—
$CDX2$	CDX2	NANOG, OCT4
$NANOG$	PBX1, OCT4-SOX2,KLF4	NANOG, GATA6
$GATA6$	GATA6, OCT4-SOX2	NANOG, OCT4
$GCNF$	CDX2, GATA6	—
$KLF4$	NANOG, OCT4, SOX2	—
$OCT4$	OCT4-SOX2	GCNF, CDX2
$SOX2$	OCT4-SOX2	—

Table S2. Interaction rules for genes in the pluripotency network
