# Supplementary material for: Rare-event sampling of epigenetic landscapes and phenotype transitions
Source: PLoS Comput Biol. 2018 Aug 3;14(8):e1006336. doi: 10.1371/journal.pcbi.1006336 (PMC6093701; doi:10.1371/journal.pcbi.1006336)
Supplement: S3 Table — (PDF) [file pcbi.1006336.s006.pdf]

**Parameter**	Set I	Set II	**Description**
g_{off}	100	100	basal/ repressed expression rate
g_{on}	3900	3900	activated expression rate
h	1×10^{-5}	5×10^{-5}	binding rate of transcription factor
f	10	50	unbinding rate of transcription factor
k	1	1	transcription factor degradation rate

Table S3. Parameters of the pluripotency network in units of k^{-1}
