# Supplementary material for: Rare-event sampling of epigenetic landscapes and phenotype transitions
Source: PLoS Comput Biol. 2018 Aug 3;14(8):e1006336. doi: 10.1371/journal.pcbi.1006336 (PMC6093701; doi:10.1371/journal.pcbi.1006336)
Supplement: S4 Table — (PDF) [file pcbi.1006336.s007.pdf]

**WE Parameters**	**ExMISA (Voronoi Movement)**	**ExMISA (Transition Matrix Mode)**	**Pluripotency f = 10 and f = 50 (Voronoi Movement)**	**Pluripotency f = 10 and f = 50 (Transition Matrix Mode)**
τ	10000	10000	10	10
Simulation regions	300	300	250	250
Replicas per region	100	100	500	500
Iterations	60	600	60	600

Table S4. Weighted Ensemble simulation parameters for all networks.
