# Supplementary material for: Rare-event sampling of epigenetic landscapes and phenotype transitions
Source: PLoS Comput Biol. 2018 Aug 3;14(8):e1006336. doi: 10.1371/journal.pcbi.1006336 (PMC6093701; doi:10.1371/journal.pcbi.1006336)
Supplement: S5 Table — (PDF) [file pcbi.1006336.s008.pdf]

ExMISA Network				
	**State 1 (lo/lo)**	**State 2 (hi/hi)**	**State3 (lo/hi)**	**State 4 (hi/lo)**
State 1 (lo/lo)	8.96×10^{-1}	5.63×10^{-4}	5.29×10^{-2}	5.05×10^{-2}
State 2 (hi/hi)	1.24×10^{-4}	9.53×10^{-1}	2.22×10^{-2}	2.45×10^{-2}
State 3 (lo/hi)	1.16×10^{-2}	1.99×10^{-2}	9.68×10^{-1}	5.52×10^{-4}
State 4 (hi/lo)	1.05×10^{-2}	2.15×10^{-2}	6.78×10^{-4}	9.67×10^{-1}

Pluripotency Network Parameter Set I						
	**State 1 (LN2)**	**State 2 (PE)**	**State3 (TE)**	**State 4 (SC)**	**State 5 (LN1)**	**State 6 (IM)**
State 1 (LN2)	2.97×10^{-1}	2.50×10^{-1}	6.04×10^{-2}	3.47×10^{-3}	2.68×10^{-1}	2.07×10^{-2}
State 2 (PE)	1.42×10^{-3}	8.90×10^{-1}	3.06×10^{-4}	1.83×10^{-4}	1.11×10^{-2}	9.66×10^{-2}
State 3 (TE)	1.91×10^{-4}	2.11×10^{-4}	8.03×10^{-1}	3.03×10^{-7}	1.00×10^{-4}	1.96×10^{-1}
State 4 (SC)	5.09×10^{-12}	4.30×10^{-6}	9.34×10^{-6}	4.30×10^{-1}	5.70×10^{-1}	1.39×10^{-7}
State 5 (LN1)	2.06×10^{-6}	5.16×10^{-6}	3.53×10^{-5}	5.16×10^{-2}	9.48×10^{-1}	8.20×10^{-6}
State 6 (IM)	2.72×10^{-7}	4.14×10^{-4}	1.64×10^{-3}	1.01×10^{-9}	1.36×10^{-6}	9.98×10^{-1}

Pluripotency Network Parameter Set II					
	**State 1 (TE)**	**State 2 (PE)**	**State3 (SC)**	**State 4 (LN)**	**State 5 (IM)**
State 1 (TE)	8.05×10^{-1}	2.66×10^{-6}	1.31×10^{-6}	2.92×10^{-3}	1.92×10^{-1}
State 2 (PE)	3.09×10^{-7}	9.20×10^{-1}	3.13×10^{-7}	1.65×10^{-4}	7.98×10^{-2}
State 3 (SC)	4.70×10^{-8}	2.21×10^{-7}	8.23×10^{-1}	1.77×10^{-7}	2.38×10^{-6}
State 4 (LN)	5.25×10^{-9}	4.92×10^{-8}	6.86×10^{-3}	9.93×10^{-1}	5.02×10^{-7}
State 5 (IM)	1.60×10^{-9}	8.22×10^{-9}	2.22×10^{-9}	1.01×10^{-8}	9.99×10^{-1}

Table S5. Transition Matrices of Metastable Phenotype Clusters (MSMs). Markov State Models of metastable phenotype-cluster transitions found through the computational pipeline for all three simulated networks. There are four different combinations of a/b protein expression levels in the coarse-grained phenotype network of the ExMISA network: lo/lo, hi/hi, lo/hi, and hi/lo. The steady-state probabilities of the lo/lo, hi/hi, lo/hi, and hi/lo cell phenotypes are predicted by the computational pipeline to be 1.71×10^{-2}, 7.67×10^{-2}, 3.71×10^{-1}, 3.80×10^{-1}, respectively. The steady state probabilities of the six and five coarse-grained phenotype networks in the pluripotency network Parameter Set I and Parameter Set II can be found in figures 4 and 6, respectively.
