# Supplementary material for: Rare-event sampling of epigenetic landscapes and phenotype transitions
Source: PLoS Comput Biol. 2018 Aug 3;14(8):e1006336. doi: 10.1371/journal.pcbi.1006336 (PMC6093701; doi:10.1371/journal.pcbi.1006336)
Supplement: S6 Table — (PDF) [file pcbi.1006336.s009.pdf]

Method	Start/End State	MFPT	5% Confidence	95% Confidence
CME - numeric benchmark	Basin centers	1.84×10^5	—	—
Conventional SSA simulation	Basin centers	1.82×10^5	1.67×10^5	1.98×10^5
WE - rate mode	Basin centers	1.82×10^5	1.78×10^5	1.85×10^5
WE - transition matrix mode	Coarse-grained polarized phenotype	2.34×10^5	—	—
Coarse-grained phenotype network	Coarse-grained polarized phenotype	1.70×10^5	—	—

Table S6. Computed Mean First Passage Times in the ExMISA Network–Comparison of Different Methods. Computed Mean First Passage Times (MFPTs, time-units k^{-1}) of the ExMISA network, using different computation methods. For each row, $\mathrm{MFPT}_{XY} = \mathrm{MFPT}_{YX}$ due to symmetry in the network, and the start- and end-state (X and Y) for the transition are defined either with respect to distance from the centers of the polarized phenotype basins, or in terms of aggregated states in the coarse-grained phenotype definition. For basin centers, State X is defined as a hypersphere of radius 1 centered around the state vector [4,16,0,0,1,0,1,0], corresponding to the species: [a,b,A_{00},A_{10},A_{01},B_{00},B_{10},B_{01}]. State Y is a hypersphere centered around [16,4,0,1,0,0,0,1]. For the coarse-grained phenotype definition, states correspond to the polarized a/b hi/lo and lo/hi phenotypes.
