# Supplementary material for: Rare-event sampling of epigenetic landscapes and phenotype transitions
Source: PLoS Comput Biol. 2018 Aug 3;14(8):e1006336. doi: 10.1371/journal.pcbi.1006336 (PMC6093701; doi:10.1371/journal.pcbi.1006336)
Supplement: S7 Table — (PDF) [file pcbi.1006336.s010.pdf]

**Method**	**Transition**			
	**SC → LN1**	**LN1 → SC**	**SC → TE**	**TE → SC**
CME	—	—	—	—
ConventionalSSA	$1.85(1.75, 2.33) \times 10^1$	$2.69(2.30, 3.43) \times 10^2$	$1.65(1.07, 2.34) \times 10^5$	$3.59(2.50, 5.18) \times 10^5$
Weighted Ensemble Rate Mode	$1.71(1.64, 1.78) \times 10^1$	$1.94(1.85, 2.05) \times 10^2$	$1.36(1.02, 1.77) \times 10^5$	$2.70(2.48, 2.91) \times 10^5$
Weighted Ensemble Transition Matrix Mode	2.09×10^1	4.13×10^2	2.19×10^5	2.21×10^5
MSM	2.23×10^1	3.89×10^2	2.06×10^5	2.20×10^5

Table S7. Computed Mean First Passage Times of Inter-Phenotype Transitions in the Pluripotency Network (Parameter Set I). The Mean First Passage Times of NANOG Fluctuation and (De)differentiation in the pluripotency network calculated using $\tau = 10$ and $f = 10$. The MFPT reported for the WE is a block average over the last 500 iterations. The MFPT and standard deviation are found for transitioning between the stem cell phenotype (SC) and the pluripotent phenotype with low NANOG expression (LN1) (analogous to high NANOG production (N^{hi}) and low NANOG production (N^{lo}) transitions measured in experiments) and for transitioning between the stem cell phenotype (SC) and the trophectoderm phenotype (TE), calculated on the timescale of the protein degradation rate k. The SC, TE, and LN1 regions of interest (ROI) are defined as the SC, TE, and LN1 phenotypes derived from the MSM reduction of the sampled transition matrix.
