# Supplementary material for: Rare-event sampling of epigenetic landscapes and phenotype transitions
Source: PLoS Comput Biol. 2018 Aug 3;14(8):e1006336. doi: 10.1371/journal.pcbi.1006336 (PMC6093701; doi:10.1371/journal.pcbi.1006336)
Supplement: S1 Fig — (PDF) [file pcbi.1006336.s011.pdf]

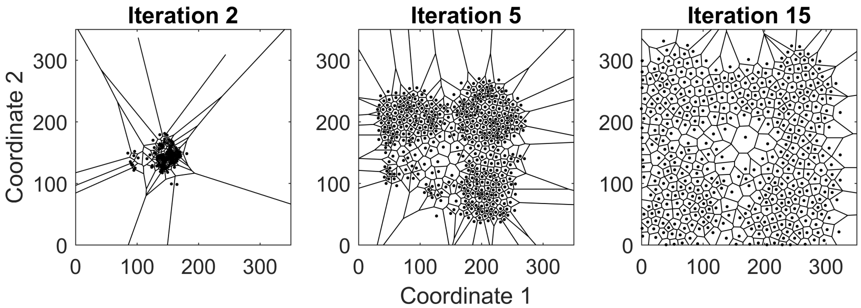

Fig 1. Movement of Voronoi Centers during weighted ensemble sampling. Starting from the left are shown three successive iterations of the adaptive WE simulation for a representative network.
