# Supplementary material for: Rare-event sampling of epigenetic landscapes and phenotype transitions
Source: PLoS Comput Biol. 2018 Aug 3;14(8):e1006336. doi: 10.1371/journal.pcbi.1006336 (PMC6093701; doi:10.1371/journal.pcbi.1006336)
Supplement: S2 Fig — (PDF) [file pcbi.1006336.s012.pdf]

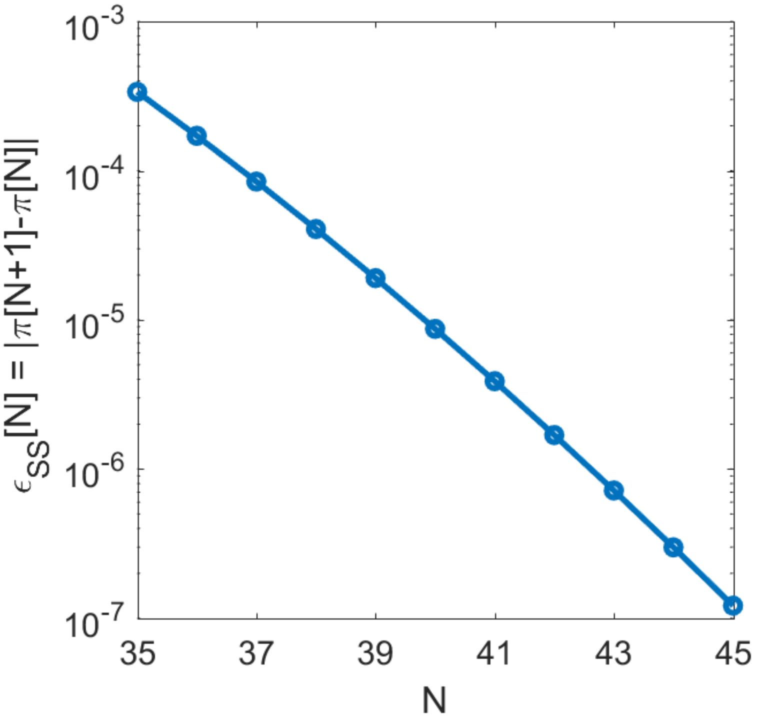

Fig 1. Error in computed steady-state probability as a function of N, the number of protein states retained in the state-space truncation. N corresponds to the maximum allowed copy-number of transcription factors a and b in the ExMISA network. For a truncation to N, probability flux between states with n_a, $n_b \leq N$ and states with n_a, $n_b > N$ is assumed to be 0 (i.e., the boundaries of the state-space are reflective). The error $\epsilon_S S[N]$ is defined by $\sum_i |\pi[N+1] - \pi[N]|$, where i runs over all enumerated states of the state-space with truncation to $N+1$ (all states outside the boundary have probability 0). That is, the error is computed as the sum of the absolute difference between steady-state probabilities for each state, comparing $\pi[N]$ (steady-state probability computed with truncation to N) to $\pi[N+1]$ (truncated to $N+1$).
