# Supplementary material for: Rare-event sampling of epigenetic landscapes and phenotype transitions
Source: PLoS Comput Biol. 2018 Aug 3;14(8):e1006336. doi: 10.1371/journal.pcbi.1006336 (PMC6093701; doi:10.1371/journal.pcbi.1006336)
Supplement: S3 Fig — (PDF) [file pcbi.1006336.s013.pdf]

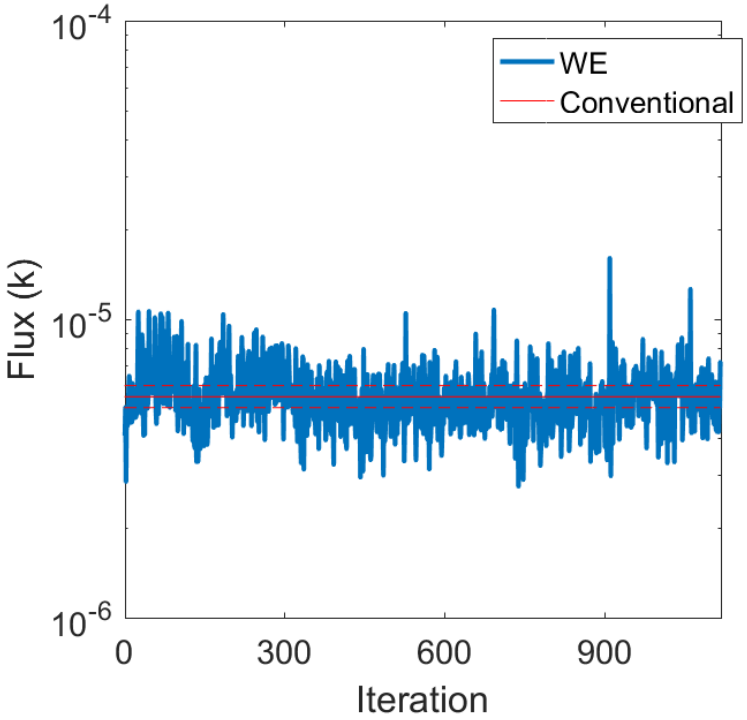

Fig 1. Convergence of the flux of the transition between the polarized phenotype-states in the ExMISA network. The 5% and 95% confidence intervals for the long conventional simulation are shown in dotted red lines. The flux between the a/b hi/lo and lo/hi phenotypes was calculated using WE sampling with parameters: $\tau = 200$, 300 bins, and 50 replicas per bin. The system was sampled for 1100 iterations of τ.
