# Supplementary material for: Rare-event sampling of epigenetic landscapes and phenotype transitions
Source: PLoS Comput Biol. 2018 Aug 3;14(8):e1006336. doi: 10.1371/journal.pcbi.1006336 (PMC6093701; doi:10.1371/journal.pcbi.1006336)
Supplement: S4 Fig — (PDF) [file pcbi.1006336.s014.pdf]

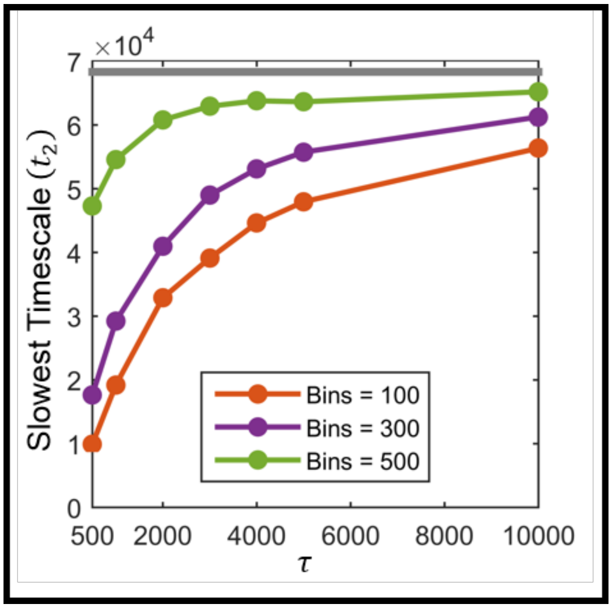

Fig 1. Convergence of the slowest implied timescale t_2 with increasing number of sampling regions (bins) and increasing lagtime τ. The lagtime calculated using the truncated CME is shown in gray. The accuracy of the WE approximation increases monotonically with increasing bin number and lagtime.
