# Supplementary material for: Rare-event sampling of epigenetic landscapes and phenotype transitions
Source: PLoS Comput Biol. 2018 Aug 3;14(8):e1006336. doi: 10.1371/journal.pcbi.1006336 (PMC6093701; doi:10.1371/journal.pcbi.1006336)
Supplement: S5 Fig — (PDF) [file pcbi.1006336.s015.pdf]

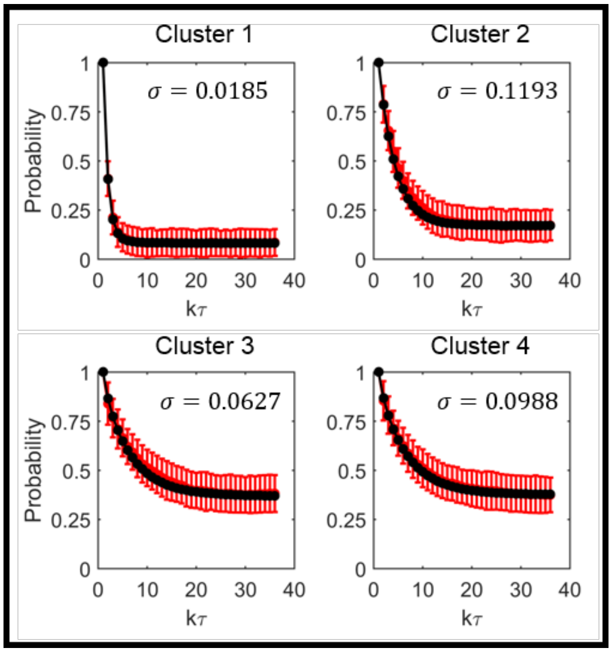

Fig 1. The Chapman-Kolmogorov test on the four Markov State Model phenotypes of the sampled ExMISA network. The relaxation curves and variance of a 1000τ trajectory are shown in red. The relaxation curve predictions from the MSM transition matrix is shown in black. The total error σ is measured as the 2-norm containing the differences between the two estimates of the relaxation curve.
