# Supplementary material for: Rare-event sampling of epigenetic landscapes and phenotype transitions
Source: PLoS Comput Biol. 2018 Aug 3;14(8):e1006336. doi: 10.1371/journal.pcbi.1006336 (PMC6093701; doi:10.1371/journal.pcbi.1006336)
Supplement: S7 Fig — (PDF) [file pcbi.1006336.s017.pdf]

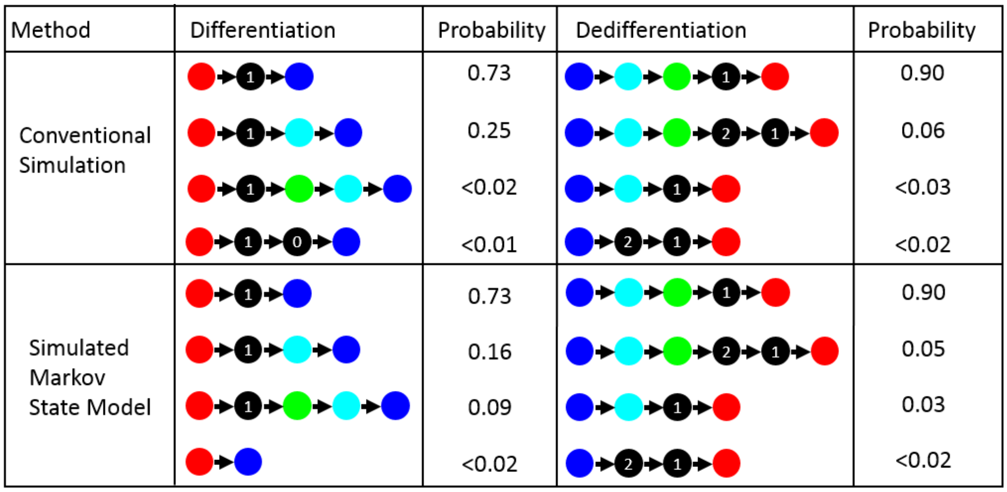


**Fig 1. Validation of the SC → TE transition pathway calculated through weighted ensemble sampling.** The parallel transition pathways are compared against those calculated from a single long conventional simulation.
