# Supplementary material for: Rare-event sampling of epigenetic landscapes and phenotype transitions
Source: PLoS Comput Biol. 2018 Aug 3;14(8):e1006336. doi: 10.1371/journal.pcbi.1006336 (PMC6093701; doi:10.1371/journal.pcbi.1006336)
Supplement: S8 Fig — (PDF) [file pcbi.1006336.s018.pdf]

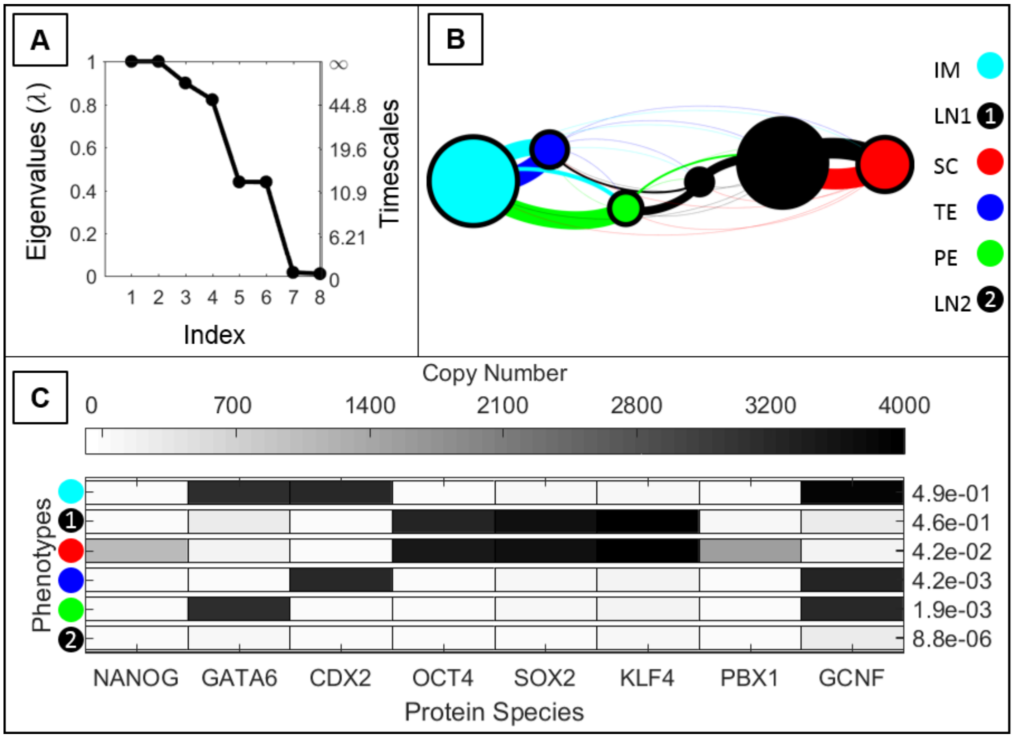

Fig 1. Reproducibility of the weighted ensemble sampling of the pluripotency network. The second WE sampling of $f = 10$ parameter set was initialized in the same manner as the first. A) Eigenvalues and timescales. B) MSM C) Macrostate compositions
