# Supplementary material for: Rare-event sampling of epigenetic landscapes and phenotype transitions
Source: PLoS Comput Biol. 2018 Aug 3;14(8):e1006336. doi: 10.1371/journal.pcbi.1006336 (PMC6093701; doi:10.1371/journal.pcbi.1006336)
Supplement: S9 Fig — (PDF) [file pcbi.1006336.s019.pdf]

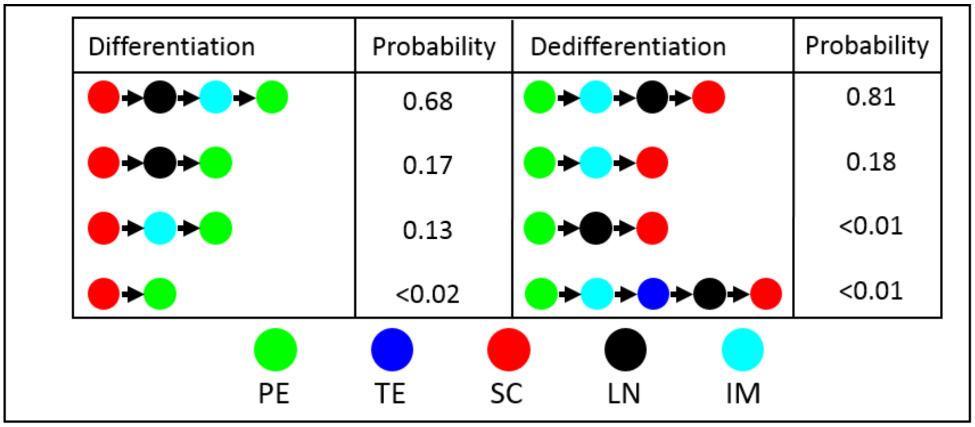

Fig 1. Pathway decomposition for the SC → PE transition for $f = 50$
