# Supplementary material for: Rare-event sampling of epigenetic landscapes and phenotype transitions
Source: PLoS Comput Biol. 2018 Aug 3;14(8):e1006336. doi: 10.1371/journal.pcbi.1006336 (PMC6093701; doi:10.1371/journal.pcbi.1006336)
Supplement: S10 Fig — (PDF) [file pcbi.1006336.s020.pdf]

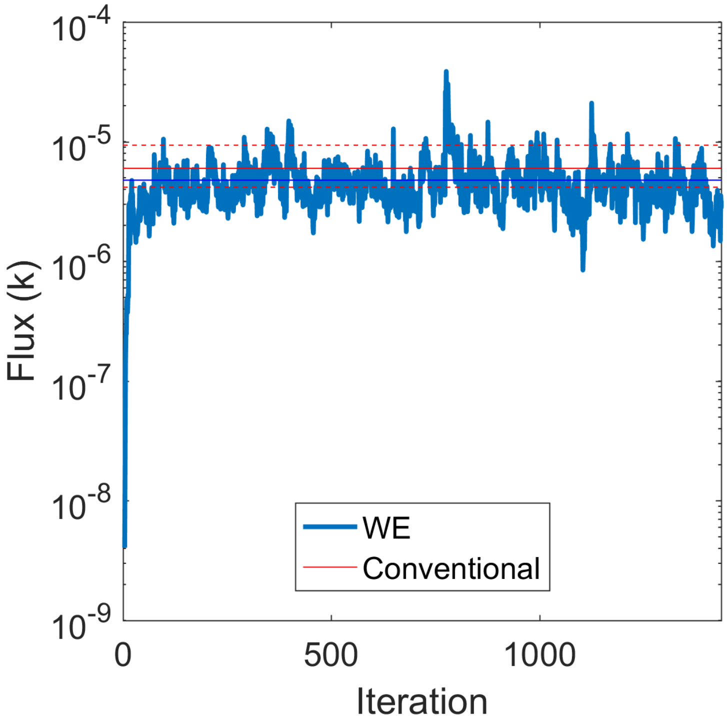

Fig 1. Convergence of the flux of the TE → SC transition in the pluripotency network with $f = 10$. Convergence of the flux of the TE → SC transition in the pluripotency network with f = 10. The 5% and 95% confidence intervals for the long conventional simulation are shown in dotted red lines. The WE flux averaged over the last 1000 iterations is shown as a solid blue line. The flux was calculated using WE sampling with parameters: $\tau = 50$, 250 bins, and 100 replicas per bin. The system was sampled for 1400 iterations of τ. The mean of the flux calculated via WE sampling is below that estimated from conventional simulation, but is within the [5, 95] confidence interval.
