# Supplementary material for: Rare-event sampling of epigenetic landscapes and phenotype transitions
Source: PLoS Comput Biol. 2018 Aug 3;14(8):e1006336. doi: 10.1371/journal.pcbi.1006336 (PMC6093701; doi:10.1371/journal.pcbi.1006336)
Supplement: S11 Fig — (PDF) [file pcbi.1006336.s021.pdf]

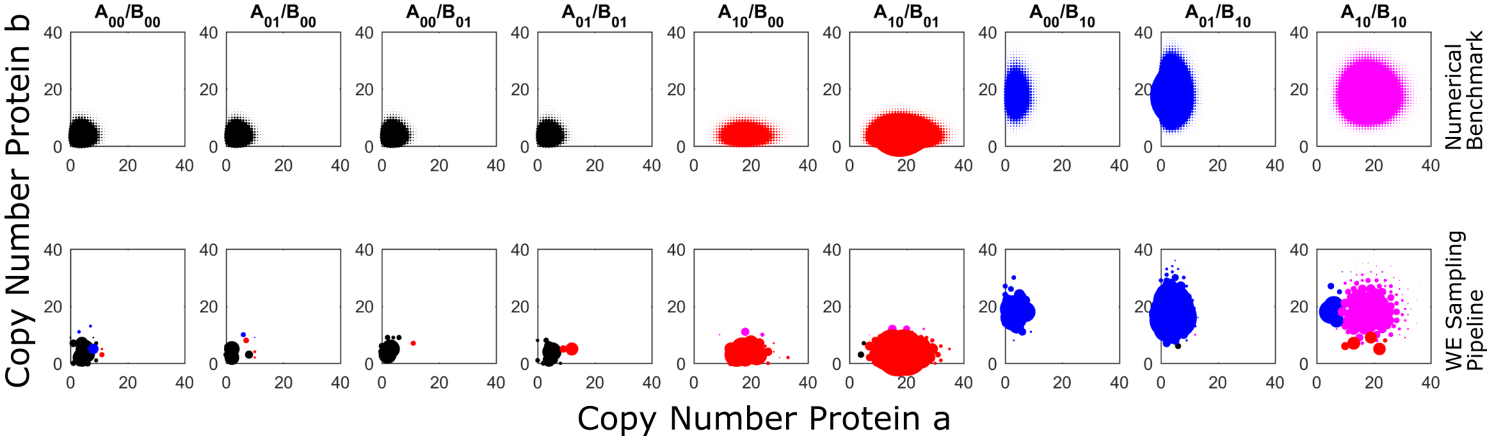

Fig 1. Difference in Coarse-Grained clustering for the 2-gene ExMISA cell decision network studied through the numerical benchmark (top) and the WE sampling pipeline (bottom). The color of each coarse-grained phenotype cluster corresponds to the expression level of protein a/b: lo/lo (black), hi/lo (red), lo/hi (blue), hi/hi (magenta). All enumerated state phenotypes are sized proportionally to their probability for each of the nine gene configurations for the numerical benchmark, while only the centers of each sampling region are shown for the WE sampling computational pipeline. While the centers of the sampling regions are mostly well separated according to their gene configuration, the phenotype states assigned to the sampling region extend across multiple gene configurations due to the choice of euclidean distance metric in assigning phenotype states to sampling regions.
