# Supplementary material for: Rare-event sampling of epigenetic landscapes and phenotype transitions
Source: PLoS Comput Biol. 2018 Aug 3;14(8):e1006336. doi: 10.1371/journal.pcbi.1006336 (PMC6093701; doi:10.1371/journal.pcbi.1006336)
Supplement: S12 Fig — (PDF) [file pcbi.1006336.s022.pdf]

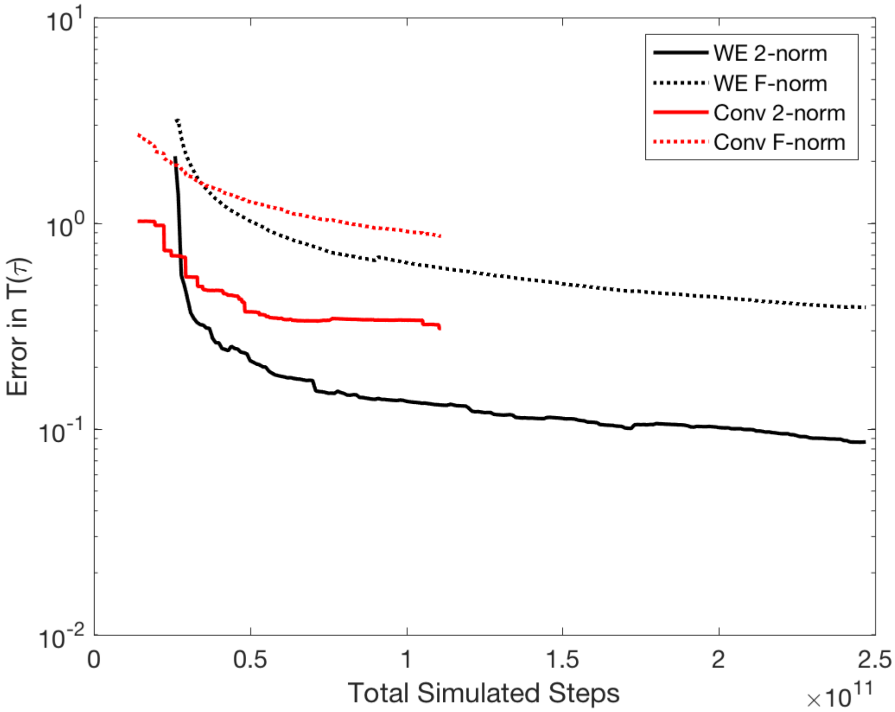

Fig 1. Plotted Errors in Sampled $\widetilde{\mathbf{T}}(\tau)$ for ExMISA. Errors for both conventional SSA simulation and the WE pipeline were computed using either the 2-norm or the Frobenius norm of $\widetilde{\mathbf{T}}(\tau)_{sim} - \widetilde{\mathbf{T}}(\tau)_{theor}$ (i.e., the difference between the simulated transition matrix and the theoretical one, which was computed numerically.) Initially, the WE simulation requires more time initially, due to the inclusion of the iterations used for the adaptive movement of Voronoi regions. By 5×10^{10} steps, the WE performance has surpassed that of Conventional SSA. Note that the inherent parallelization of the WE algorithm made it possible to achieve more total simulation steps in WE versus Conventional SSA.
